# The WHO 2021 Classification of Central Nervous System tumours: a practical update on what neurosurgeons need to know—a minireview

**DOI:** 10.1007/s00701-022-05301-y

**Published:** 2022-07-26

**Authors:** Sverre Helge Torp, Ole Solheim, Anne Jarstein Skjulsvik

**Affiliations:** 1 Department of Clinical and Molecular Medicine, Faculty of Medicine and Health Sciences, Laboratory Centre, NTNU - Norwegian University of Science and Technology, St. Olavs Hospital, NO-7491 Trondheim, Norway; 2 Department of Pathology, St. Olavs Hospital, Trondheim University Hospital, Trondheim, Norway; 3 Department of Neurosurgery, St. Olavs Hospital, Trondheim University Hospital, Trondheim, Norway; 4 Department of Neuromedicine and Movement Science, Faculty of Medicine and Health Sciences, NTNU - Norwegian University of Science and Technology, Trondheim, Norway

**Keywords:** Brain tumours, Biomarker, Diagnosis, Pathology, Molecular genetics

## Abstract

**Background:**

The World Health Organization (WHO) Classification of Tumours, also known as WHO Blue Books, represents an international standardised tool in the diagnostic work-up of tumours. This classification system is under continuous revision, and progress in the molecular classification of tumours in the central nervous system (CNS) enforced an update of the WHO 2016 classification, and the fifth edition, WHO CNS5, was published in 2021. The aim of this minireview is to highlight important changes in this new edition relevant for the practicing neurosurgeon.

**Methods:**

The sixth volume of the fifth edition of the WHO Blue Books of CNS tumours and related papers formed the basis for this minireview.

**Results:**

Major changes encompass standardisation of tumour grading and nomenclature as well as increased incorporation of molecular markers in the classification of CNS tumours.

**Conclusion:**

Advances in molecular genetics have resulted in more accurate diagnosis and prognosis of CNS tumours, and this minireview summarises important changes implemented in the last edition of WHO classification of CNS tumours important for the practicing neurosurgeon.

## Introduction

The famous German pathologist Rudolf Virchow (1821–1902), the founder of modern pathology, was the first to link the origin of cancers from otherwise normal cells [42]. Later the histopathological diagnosis of tumours was to a large extent made on comparing tumour cell features with those of normal tissue, as for astrocytomas, brain tumours with cells resembling astrocytes were called astrocytomas. This approach was systematically employed in the book by Bailey and Cushing from 1926, *A Classification of the Tumours of the Glioma Group on a Histogenetic Basis with a Correlated Study of Prognosis* [1, 29]. Here, the concept of tumours arising from immature precursor cells was proposed as well. Then, grading of gliomas based on cytological criteria was launched by Kernohan in 1949 [18] and Ringertz in 1950 [36]. This concept is fundamental even today and led to a classification and grading system for central nervous system (CNS) tumours launched by the World Health Organization (WHO), with the first edition presented by Zülch et al. in 1979 [46]. This classification was primarily based on light microscopic changes in haematoxylin eosin–stained sections, later immunostainings and electron microscopic changes were included as well. This system has become the international standard for diagnostics of CNS tumours with periodic revisions in 1993, 2000, 2007 and 2016.

During the last decade, there has been a paradigm shift in CNS tumour diagnostic as advances in molecular genetics have revealed alterations in these tumours. Already in the 2016 WHO classification, molecular alterations were introduced in the diagnostic work-up of some tumours with establishment of an integrated and layered diagnosis in which histopathology and molecular information were included [25, 26]. Further progress of molecular classification of CNS tumours prompted a need for an update and lead to the foundation of cIMPACT-NOW (the Consortium to Inform Molecular and Practical Approaches to CNS Tumour Taxonomy – Not Official WHO) with the aim to communicate recent discoveries important for clinical practice in advance to the now released WHO 2021 edition (WHO CNS5) where molecular data are implemented to a large extent [9, 23, 27, 28]. In any case, WHO CNS5 represents a work in progress as advances in molecular genetics will form the basis for a continuous revision of CNS tumour classification.

This summary of WHO CNS5 highlights relevant clinicopathological updates on the most common CNS tumours relevant to neurosurgeons. For more detailed information, the reader is referred to the WHO CNS5 classification available electronic or in a printed version (WHO Blue book) [45] as well as to related papers, amongst which some are found in the Reference list.

## General updates

The classification of CNS tumours in WHO CNS5 follows to a large extent that of WHO 2016; however, the chapter of gliomas and neuronal/neuronal-glial tumours has undergone major revision due to progress in molecular genetics. Furthermore, tumour types common to other organ systems are grouped together: mesenchymal (non-meningothelial) tumours, melanocytic tumours etc. A chapter on genetic tumour syndromes is also added. Thus, WHO CNS5 incorporates to a larger extent molecular genetics with clinical relevance, so this last edition comprises elements from both histopathology and molecular genetics giving rise to a somewhat mixed taxonomy [27]. Table 1 shows the groups of CNS tumours in the 2021 WHO classification.

**Table 1:** The WHO CNS5 groups of tumours

1. Gliomas, glioneuronal and neuronal tumours
2. Choroid plexus tumours
3. Embryonal tumours
4. Pineal tumours
5. Cranial and paraspinal nerve tumours
6. Meningiomas
7. Mesenchymal, non-meningothelial tumours involving the CNS
8. Melanocytic tumours
9. Haematolymphoid tumours involving the CNS
10. Germ cell tumours
11. Tumours of the sellar region
12. Metastases to the CNS
13. Genetic tumour syndromes involving the CNS

Histopathological features have traditionally formed the basis for characterisation and grading of CNS tumours; however, such features may be heterogenous within a given tumour resulting in potential sampling error and underestimating a tumour’s biological behaviour. Molecular genetic changes appear more uniform, giving a lower possibility of molecular undersampling, even in small biopsy specimens [6]. Accordingly, molecular information has become important to achieve greater diagnostic accuracy, more precise prognosis and optimised patient management and treatment options, important elements of personalised medicine. This reinforces the use of a “layered report structure” in which histopathology, grading and molecular information are combined to form an integrated diagnosis, as shown in Table 2, including an example [25, 27]. In this regard, it is worth mentioning that the revised classification of many of the CNS tumours based on molecular alterations must be kept in mind when data from older clinical trials should be used to the newly defined types [2]. Accordingly, data from prior studies cannot merely be transferred to current trials.

**Table 2:** Layered diagnosis with an example

Layer	Example
Layer 1 Integrated diagnosis	Diffuse astrocytoma, *IDH*-mutant, CNS WHO grade 2
Layer 2 Histopathological diagnosis	Diffuse astrocytoma
Layer 3 WHO grade	CNS WHO grade 2
Layer 4 Molecular genetics	IDH1 R132H-mutant, *ATRX*-mutant, *TP53*-mutant

Regarding taxonomy, “type” replaces “entity” and “subtype” replaces “variant”, and some diagnoses have been revised for clarity, as “anaplastic” is removed, so diagnoses such as “anaplastic astrocytoma” and “anaplastic oligodendroglioma” are omitted (but are still kept for *anaplastic meningiomas*). In addition, anatomical sites are deleted in some tumours, as for *chordoid glioma* (“of the third ventricle” is omitted) [27].

In WHO CNS5, the guidelines in reporting gene symbols, gene names and chromosomal alterations are updated and standardised, for instance, gene symbols are written in italics whereas proteins and gene groups are not italicised [27]. Furthermore, the units of lengths have been changed, so tumour size shall now be given in millimetres (mm) rather than centimetres (cm) to avoid the use of decimals [10].

Regarding tumour grading, Arabic numerals are now used instead of Roman ones (grading of some CNS tumours is shown in Table 3). Grading shall now also be done *within* tumour types rather than *across* tumour types, for instance, *astrocytoma IDH-mutant* can now be either grade 2, 3 or 4 [16]. Since grading of CNS tumours may differ somewhat from tumours in other organs, it is recommended to use the term “CNS WHO grade” [27]. Grading is primarily based on a tumour’s natural biology without any treatment. This can, however, be problematic to estimate because most patients today receive treatment that influences the disease course. For instance, *WNT-activated medulloblastoma*, a highly malignant tumour without treatment, is recorded as CNS WHO grade 4, but responds well on current treatment regimes. Tumour grading is traditionally based on a sum of atypical histopathological features; however, some molecular biomarkers have been shown to have stronger prognostic power than histopathology, and as such more accurately identify patients at higher risk of recurrence—*molecular beats histology* [2].

**Table 3:** Grading of some tumours according to CNS WHO5

Tumour type	Grade
Astrocytoma,* IDH*-mutant	2, 3, 4
Oligodendroglioma, *IDH*-mutant and 1p/19q-codeled	2, 3
Glioblastoma, *IDH*-wildtype	4
Diffuse midline glioma, H3 K27-altered	4
Diffuse hemispheric glioma, H3 G34-mutant	4
Pilocytic astrocytoma	1
Pleomorphic xanthoastrocytoma	2, 3
Embryonal tumours	4
Myxopapillary ependymoma	2
Meningioma	1, 2, 3
Solitary fibrous tumour	1, 2, 3

Several methods are used in molecular testing; however, WHO CNS5 does not recommend any specific methods [27]. In molecular characterising of CNS tumours, next generation sequencing (NGS) gene panels for brain tumours and methylation profiling have become very useful and efficient [22, 39]. A tumour’s molecular signature may in some instances give the rationale for targeted therapy, as BRAF-targeted therapy has shown positive response in certain brain tumours with *BRAF V600 *mutation [21]. Furthermore, methylation profiling has been successful for several tumour types and has in many cases proven to be more specific than conventional histopathology [8, 38]. In WHO CNS5, information about the methylation profile is given for most of the tumours. Molecular diagnostics will be increasingly incorporated in the classification of CNS tumours; however, molecular genetic analyses may delay time-to-diagnosis and subsequently treatment. In that regard, novel NGS technique such as Nanopore sequencing will allow for more rapid diagnostic testing [33]

The assessment of mitotic counts has been changed in the last WHO edition from number of mitoses per 10 high power fields (HPF) to a defined area in mm^2^ (requiring adjustments to individual microscopes) [10]. Ki-67/MIB-1 proliferating index is mentioned for many tumours and appears as a useful biomarker in grading and prognostications; however, as stated earlier, differences in techniques and determination make it problematic to establish reliable cutoff values [34].

The definition and application of *NOS* (“not otherwise specified”) and *NEC* (“not elsewhere classified”) are more precisely defined in WHO CNS5. The *NOS* suffix means that molecular information is insufficient or not available to make a specific diagnosis. The use of *NEC* encompasses that adequate analyses have been undertaken but the results do not provide a precise diagnosis within the WHO classification scheme, often then a more descriptive diagnosis may be given [9].

## Specific tumour updates

### Gliomas, glioneuronal and neuronal tumours.

Diffuse gliomas are now divided into those occurring primarily in adults (“adult-type”) or in children (“paediatric type”) (see Table 4). With “primarily” means that paediatric tumours may occur in adults, especially young adults, and adult tumours may rarely appear in children [27].

**Table 4:** WHO CNS5 classification of gliomas, glioneuronal and neuronal tumours

Tumour group	Types
Adult-type diffuse gliomas	- Astrocytoma, *IDH*-mutant - Oligodendroglioma, *IDH*-mutant and 1p/19q-codeleted - Glioblastoma, *IDH*-wildtype
Paediatric-type diffuse low-grade gliomas	- Diffuse astrocytoma, MYB- or MYBL1-altered - Angiocentric glioma - Polymorphous low-grade neuroepithelial tumour of the young - Diffuse low-grade glioma, MAPK pathway-altered
Paediatric-type diffuse high-grade gliomas	- Diffuse midline glioma, H3 K27-altered - Diffuse hemispheric glioma, H3 G34-mutant - Diffuse paediatric-type high-grade glioma, H3-wildtype and *IDH*-wildtype - Infant-type hemispheric glioma
Circumscribed astrocytic gliomas	- Pilocytic astrocytoma - High-grade astrocytoma with piloid features - Pleomorphic xanthoastrocytoma - Subependymal giant cell astrocytoma - Chordoid glioma - Astroblastoma, MN1-altered
Glioneuronal and neuronal tumours	- Ganglioglioma - Desmoplastic infantile ganglioglioma/desmoplastic infantile astrocytoma - Dysembryoplastic neuroepithelial tumour - Diffuse glioneuronal tumour with oligodendroglioma-like features and nuclear clusters - Papillary glioneuronal tumour - Rosette-forming glioneuronal tumour - Myxoid glioneuronal tumour - Diffuse leptomeningeal glioneuronal tumour - Gangliocytoma - Multinodular and vacuolating neuronal tumour - Dysplastic cerebellar gangliocytoma (Lhermitte-Duclos disease) - Central neurocytoma - Extraventricular neurocytoma - Cerebellar liponeurocytoma
Ependymomas	- Supratentorial ependymoma - Supratentorial ependymoma (ZFTA or YAP1 fusion-positive) - Posterior fossa ependymoma - Posterior fossa ependymoma (PFA or PFB group) - Pinal ependymoma - Spinal ependymoma, MYCN amplified - Myxopapillary ependymoma - Subependymoma

*Adult-type diffuse gliomas* now constitute only 3 categories: *astrocytoma IDH-mutant*; *oligodendroglioma*, *IDH-mutant and 1p/19-codeleted* and *glioblastoma*, *IDH-wildtype* [27].

Thus, astrocytic tumours are grouped as those with and without *IDH* mutations; those without *IDH* mutations (wildtype) are termed *glioblastomas IDH-wildtype*.

*Astrocytoma IDH-mutant* is now regarded as a single tumour type and graded as CNS WHO 2, 3 or 4 (the term “anaplastic” is now omitted) and designated *astrocytoma IDH-mutant CNS WHO grade 3*. The criteria for histopathological grading are as in WHO 2016, i.e. necrosis and/or microvascular proliferation is consistent with grade 4 and referred to as *astrocytoma IDH-mutant CNS WHO grade 4* [27]*.* Still there is no established definition of mitotic count to distinguish grades 2 and 3 [5, 20]. Even though Ki-67/MIB-1 proliferative index significantly increases with tumour grade, no cutoff value to reliably identify patients with increased risk of recurrence has been established [7, 20]. *CDKN2A/B* homozygous deletion in *IDH*-*mutant* astrocytomas has been shown to be a negative prognostic marker, and they should be diagnosed as *astrocytoma IDH-mutant*, *CNS WHO grade 4* despite lack of microvascular proliferation or necrosis [5, 27].

*Oligodendrogliomas* are diffuse gliomas characterised by *IDH* mutations and loss of chromosome 1p and 19q (1p/19q codeletion), thus assigned *oligodendroglioma*, *IDH-mutant and 1p/19q-codeleted*. Therefore, all *IDH-mutant* diffuse gliomas should be tested for 1p/19q codeletion, and diffuse gliomas with astrocytic appearance and 1p/19q codeletion are diagnosed as oligodendrogliomas [24]*.* However, the diagnosis of an *IDH-mutant oligodendroglioma* can be made as well without 1p/19q testing if immunohistochemical analyses reveal clear loss of ATRX expression and/or diffuse expression of TP53 [24]. Typical for oligodendrogliomas are also *TERTp* mutations (rare in diffuse astrocytomas) [19]. In WHO CNS5, malignancy grading of oligodendroglioma has been retained, even though the criteria to distinguish grades are not well-defined; however, brisk mitotic activity, microvascular proliferation and necrosis are associated with poorer prognosis [35]. Neither Ki-67/MIB-1 proliferative index provides reliable threshold values to risk stratify patients [35]. As in WHO 2016, the older entity “oligoastrocytoma” is out of use.

*Glioblastoma IDH-wildtype CNS WHO grade 4* typically presents necrosis and/or microvascular proliferation. It has also been observed that *IDH-wildtype* astrocytomas regarded as grades 2 or 3 based on histopathological criteria (i.e. no necroses or microvascular proliferation) behaved much as glioblastomas. For this reason, molecular alterations that could predict aggressive behaviour were assessed, including *EGFR* amplification, *TERTp* mutations, gain of chromosome 7 and loss of chromosome 10 [6]. Accordingly, an *IDH-wildtype* diffuse astrocytoma with at least one of these molecular features allows for a diagnosis of *glioblastoma IDH-wildtype CNS WHO grade 4* even in the absence of histopathology of a glioblastoma [6]. These tumours also cluster closely in DNA methylation analyses [6]. Therefore, these diffuse astrocytomas should undergo molecular testing for these genetic events to clarify whether there is a glioblastoma or not [16]. As such, diffuse astrocytoma, *IDH-wildtype*, CNS WHO grades 2 or 3 (i.e. without molecular features of glioblastoma), is rare and is no longer regarded as a tumour type in CNS WHO5 [16]. If the molecular signature is not consistent with a glioblastoma, one should consider testing for *BRAF* alterations, histone mutations (H3 K27- and H3 G34-mutant diffuse gliomas) or methylation profiling [6, 16]. In addition, *IDH-wildtype* gliomas should also be tested for H3 K27 and H3 G34 mutations [6, 43]. Patients ≥ 55 years at diagnosis with no immunoreactivity for IDH1 R132H can be diagnosed as *glioblastoma IDH-wildtype CNS WHO grade 4* if histopathological features of glioblastomas are present, the tumour is not located in the midline and there is no history of earlier low-grade glioma [26]. *Gliosarcoma*, *giant cell glioblastoma* and *epithelioid cell glioblastoma* are still registered subtypes of glioblastomas. The term “*glioblastoma multiforme*” should not be used.

In this manner, low-grade diffuse astrocytomas are now characterised by the presence of *IDH* mutations, and their overall prognosis according to WHO CNS5 will therefore presumably be better than the those classified by WHO 2016. Since *IDH*-*mutant* grade 2 and 3 astrocytomas exhibit similar prognosis [26], newer studies encompass these as “diffuse low-grade astrocytomas”. Likewise, the traditional pooling of “high-grade astrocytomas” (grades 3 and 4) should be discouraged as *IDH*-*mutant* grade 3 astrocytoma differs in molecular profile and clinical behaviour compared with *IDH-wildtype* grade 4 astrocytoma (i.e. glioblastoma).

**Fig. 1:**
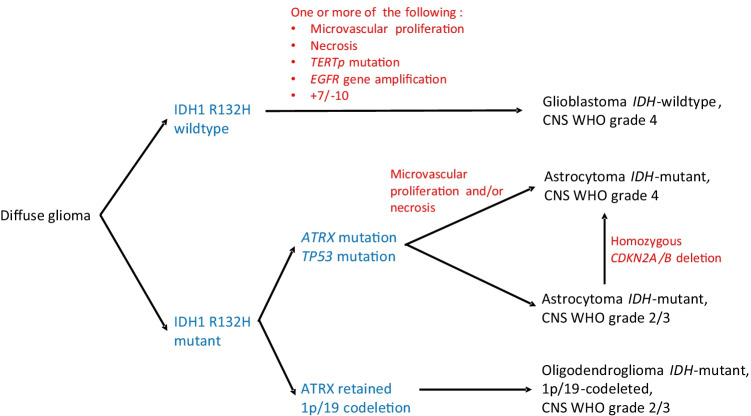
Simplified diagnostic algorithm for diffuse gliomas in adults. Astrocytoma, *IDH-wildtype* without histopathological and molecular features of glioblastoma is rare, and these tumours should undergo further molecular genetic analyses and methylation profiling. *IDH-wildtype* gliomas should also be considered for analysis of H3 K27 and H3 G34 mutations (figure inspired by [16])

Common molecular genetic events in these tumours are listed in Table 5, a simplified diagnostic algorithm is shown in Fig. 1, and Table 6 shows the updated nomenclature of gliomas.

**Table 5:** Survey of current relevant clinicopathological genetic alterations in human gliomas

Molecular marker	Clinical significance
*ATRX* mutation Alpha-thalassemia/mental retardation syndrome X	- Common in astrocytoma, *IDH*-mutant (not in oligodendroglioma) and diffuse hemispheric glioma, H3 G34–mutant
*BRAF* V600 mutation	- Frequently present in pleomorphic xanthoastrocytoma, also in ganglioglioma and epitheloid glioblastoma
*CDKN2A/B* homozygous deletion Cyclin-dependent kinase inhibitor 2A/B	- Present in astrocytoma, *IDH*-mutant indicates poor prognosis
*EGFR* gene amplification Epidermal growth factor receptor	- Common in glioblastoma, *IDH-*wildtype CNS WHO grade 4 - If present in astrocytoma, *IDH-wildtype* CNS WHO grades 2 or 3, it is consistent with glioblastoma, *IDH-wildtype* CNS WHO grade 4
*EGFR*-mutations	- Most common is *EGFR*vIII, frequently present in glioblastoma, *IDH-wildtype* CNS WHO grade 4
H3 G34 mutation Histone H3 3 G34	- Present in hemispheric diffuse glioma, *IDH-wildtype*, predominantly in children and young adults, poor prognosis
H3 K27M mutation Histone H3 K27M	- One of the criteria of diffuse midline glioma, H3 K27M altered - May occur in other gliomas not located in the midline (pilocytic astrocytoma and ependymoma)
*IDH1/2* Isocitrate dehydrogenase	- Frequently mutated in diffuse gliomas (astrocytomas and oligodendrogliomas) and is associated with better prognosis than *IDH-wildtype* gliomas
*KIAA1549-BRAF* gene fusion	- Frequently found in pilocytic astrocytoma, also in diffuse leptomeningeal glioneuronal tumour, pilomyxoid astrocytoma and ganglioglioma
*MAPK* Mitogen-activated protein kinase pathway	- Alterations typical for paediatric-type diffuse low-grade gliomas
*MGMT* promotor methylation O^6^-methylguanine DNA methyltransferase	- DNA repair enzyme, methylation predicts good response to alkylating agents such as temozolomide in glioblastoma, *IDH-wildtype*
*MYB*- or *MYBL1*-altered	- Alterations typical for a paediatric low-grade glioma
*TERTp* mutation Telomerase reverse transcriptase promotor	- Present in most oligodendroglioma - If present in diffuse astrocytoma, *IDH-wildtype* CNS WHO grades 2 and 3 (i.e. without the histopathological hallmarks of glioblastoma (necrosis and/or microvascular proliferation)), it is consistent with glioblastoma *IDH-wildtype* CNS WHO grade 4
*TP53* mutation	- Present in most astrocytoma *IDH-mutant*, rare in oligodendrogliomas
*YAP1* fusions Yes-associated protein 1	- Present in some supratentorial ependymomas, especially in paediatric tumours
*ZFTA* fusions Zinc finger translocation associated	- Present in some supratentorial ependymomas (*ZFTA:* previously named *RELA* fusions)
Gain of chromosome 7/loss of chromosome 10 (+ 7/ − 10)	- Common in glioblastoma *IDH-wildtype* CNS WHO grade 4
Loss of chromosome 1p and 19q (loss of heterozygosity) (1p/19q codeletion)	- Prerequisite for the diagnosis of oligodendroglioma

**Table 6:** Update on nomenclature of adult gliomas

WHO 2016 Classification of CNS Tumours *Diffuse astrocytic and oligodendroglial tumours*	WHO 2021 Classification of CNS Tumours *Gliomas*, *glioneuronal tumours and neuronal tumours* *Adult-type diffuse gliomas*
Diffuse astrocytoma, *IDH*-mutant, WHO grade II	Astrocytoma, *IDH*-mutant, CNS WHO grade 2
Anaplastic astrocytoma, *IDH*-mutant, WHO grade III	Astrocytoma, *IDH*-mutant, CNS WHO grade 3
Glioblastoma, *IDH*-mutant WHO grade IV (secondary glioblastoma)	Astrocytoma, *IDH*-mutant, CNS WHO grade 4
Diffuse astrocytoma, *IDH*-*wildtype*, WHO grade II*	Glioblastoma, *IDH*-*wildtype*, CNS WHO grade 4
Anaplastic astrocytoma, *IDH*-*wildtype*, WHO grade III*	
Glioblastoma, *IDH*-*wildtype*, WHO grade IV (primary glioblastoma)	
Oligodendroglioma, *IDH*-mutant and 1p/19q codeleted, WHO grade II	Oligodendroglioma, *IDH*-mutant and 1p/19q codeleted, CNS WHO grade 2
Anaplastic oligodendroglioma, *IDH*-mutant and 1p/19q codeleted WHO grade III	Oligodendroglioma, *IDH*-mutant and 1p/19q codeleted, CNS WHO grade 3

^*^Detection of *TERTp* mutation, *EGFR* amplification, and/or +7/−10 (Table inspired by [42])

*Paediatric-type diffuse gliomas* are uncommon; more frequent are circumscribed gliomas and glioneuronal tumours, such as pilocytic astrocytoma and ganglioglioma. Advances in molecular genetic analyses and methylation profiling have resulted in substantial changes in the classification of these tumours. These diffuse gliomas may display astrocytic or oligodendroglial differentiation, and they are divided into low- and high-grade tumours (see Table 4). They are all *IDH-wildtype*. The *low-grade tumours* have favourable diagnosis and correspond to CNS WHO grade 1. They are categorised based on *MYB/MYB1* and MAPK pathway alterations as well as on typical histopathology [27]. The *high-grade tumours* often have mutations in histone genes, and the prognosis is in general poor [12]. Their histopathology varies, but anaplastic features with many mitoses, high cellularity, necrosis and microvascular proliferations are common. The *diffuse midline glioma* is now more precisely defined; as it requires diffuse infiltrative growth in the brain tissue, it must affect midline structures (thalamus, brain stem and spinal cord) and have H3 K27 alterations (“H3 K27 mutant” has now been changed to “H3 K27 alterations” to encompass alternative mechanisms). This clarification is important as there are other gliomas with such mutations (pilocytic astrocytomas and ependymomas) [9, 24]. High-grade hemispheric gliomas in adolescents and young adults are often characterised by histone H3 G34 mutations [28]. In conclusion, molecular genetic analysis and/or methylation profiling are essential in the diagnostic work-up of paediatric brain tumours for proper classification and molecular targeted therapy.

*Circumscribed astrocytic gliomas* include tumours with well-defined margins and to a lesser extent infiltrative growth. *Pilocytic astrocytomas* are the most common type and retain as CNS WHO grade 1. They may be diagnosed by means of classical histopathology or by a low-grade piloid astrocytic neoplasm with a solitary *MAPK* alteration, such as *KIAA1549*::*BRAF* tandem duplication and fusion. *Pilomyxoid astrocytoma* is a subtype with a somewhat poorer prognosis but still grade 1. *Pilocytic astrocytoma with histological features of anaplasia* is another subtype and shares pheno- and genotypical features with another entity, *high-grade astrocytoma with piloid features*, both with aggressive biology [41]. *Pleomorphic astrocytoma (PXA)* has a somewhat typical histopathology and characterised by *BRAF* V600 mutations, which can be assessed by immunohistochemistry. They are graded as grade 2 or 3 dependent on mitotic counts [14].

*Glioneuronal and neuronal tumours* comprise all neuronal or mixed glial-neuronal tumours with three new types added: *diffuse glioneuronal tumour with oligodendroglioma-like features and nuclear clusters*, *multinodular and vacuolating neuronal tumour* and *myxoid glioneuronal tumour.* Regarding *gangliogliomas* and *neurocytomas*, anaplastic histopathological features may rarely be present and indicative for a more aggressive tumours; however, they are still recognised as grade 1 and 2, respectively.

*Ependymoma*s are now classified based on histopathology, location and molecular features with typical signatures related to anatomic site [11]. As far as location is concerned, there are 3 categories: supratentorial (ST), infratentorial (PF (posterior fossa) and spinal (SP) tumours. ST ependymomas are divided into *ZFTA* (zinc finger translocation-associated, previously named *RELA*) or *YAP1* (Yes-associated protein 1) fusion-positive. PF ependymomas are divided into those with absent (PFA) or present (PFB) histone H3 K27-trimethylation, the former presents poorer prognosis. Amongst SP ependymomas, those with *MYCN*-amplified have a poor clinical course. In the diagnostic work-up of ependymomas, DNA methylation profiling has become a powerful tool and distinguishes types of ependymomas of the various anatomical sites [11, 16]. Papillary, clear cell and tanycytic ependymomas are morphological subtypes of ependymomas but without clinical relevance and no longer included in the classification of ependymomas [11]. Ependymomas can also be defined by anatomical site or if molecular testing is diverging or lacking. The prognostic value of malignancy grading of ependymomas is debatable but is established practice in ST ependymomas in adults and when a molecular signature lacks; however, the term “anaplastic” is dropped [9, 11]. Both *subependymoma* and *myxopapillary ependymoma* are diagnosed based on histopathology; the latter is upgraded to grade 2 because the recurrence rate is similar to conventional spinal ependymomas [27].

### Embryonal tumours

Embryonal tumours (listed in Table 7) are all grade 4 and comprise a very heterogenous group of tumours with regard to histopathology and molecular genetics. They predominate amongst children and young adults. The term “primitive neuroectodermal tumour”, previously used to include many of these tumours, is outmoded due to progress in molecular genetics. Because of prognostic and predictive value, it is important to perform molecular analyses and/or methylation profiling of these tumours. Based on molecular data, some new tumour types have been added in WHO CNS5. *CNS embryonal tumour* denotes an embryonal tumour that needs further investigation to achieve a more specific diagnosis, i.e. they are NEC or NOS [27].

**Table 7:** Embryonal tumours

**Medulloblastoma** Medulloblastoma, molecularly defined - Medulloblastoma, WNT-activated - Medulloblastoma, SHH-activated and *TP53*-wildtype - Medulloblastoma, SHH-activated and *TP53*-mutant - Medulloblastoma, non-WNT/non-SHH Medulloblastomas, histologically defined
**Other CNS embryonal tumours** Atypical teratoid/rhabdoid tumour (AT/RT) Cribriform neuroepithelial tumour* Embryonal tumour with multilayered rosettes (ETMR) CNS neuroblastoma, *FOXR2*-activated* CNS tumour with *BCOR* internal tandem duplication* CNS embryonal tumour

^*^New tumour types added in WHO CNS5

*Medulloblastomas* are the most common amongst these tumours, and the classification is much in line with that of WHO 2016. There are 4 well-established histopathological types: *classic*, *desmoplastic/nodular*, *medulloblastoma with extensive nodularity* and *large cell/anaplastic*; however, in WHO CNS5, they are combined into one section in which these histopathological types enter into a single tumour type: *medulloblastoma*, *histologically defined* [27]. Furthermore, there are 4 molecular groups: *medulloblastoma*, *WNT-activated*, *medulloblastoma*, *SHH-activated (TP53-wildtype/mutant)*, *medulloblastoma non-WNT/non-SHH (group 3)* and *medulloblastoma non-WNT/non-SHH (group 4)*, the two latter joint as *medulloblastoma*, *non-WNT/non-SHH.* Since both histopathological and molecular types have their well-defined clinicopathological characteristics, these features should be incorporated in an integrated diagnosis [27]. The medulloblastoma subtypes often exhibit different radiological features, so the subtype can often be proposed preoperatively. The impact of surgical resection or any residual tumour varies across the subtypes; gross total resection is beneficial for Group 4 whereas this is more attenuated for the other subtypes [40].

*Atypical teratoid/rhabdoid tumour* (*AT/RT*) is typically characterised by loss of expression of the *SMARCB1* gene product integrase interactor 1 (*INI1*) protein, but three 3 molecular subtypes are also presented (*AT/RT-SHH*, *AT/RT-TYR* and *AT/RT-MYC*) with potential prognostic and predictive significance [31].

### Cranial and paraspinal nerve tumours

Cranial and paraspinal nerve tumours (shown in Table 8) may arise sporadically or in a setting of tumour predisposition syndromes, such as neurofibromatosis type 1 and 2 (NF1/2). In case of NF1, there is a proposed nomenclature for the spectrum of the related nerve tumours. Amongst schwannomas and neurofibromas, there are some subtypes; amongst the latter *atypical neurofibroma/atypical neurofibromatous* *neoplasm of uncertain biological potential* is added, which is a NF1-associated tumour with atypical histopathological features with potential to progress to malignant peripheral nerve sheath tumour. “Melanotic schwannoma” has been shown to be a well-characterised tumour and has now entered the classification as *malignant melanotic nerve sheath tumour.* Terms like “malignant schwannoma”, “neurofibrosarcoma” and “neurogenic sarcoma” are not recommended. Paraganglioma of the cauda equina/filum terminale has appeared as a distinct tumour type and is now called *cauda equina neuroendocrine tumour*, alternatively *paraganglioma of the cauda equina/cauda equina paraganglioma*. Concerning nerve tumours in peripheral nerves, one is referred to WHO Blue Books of Soft Tissue and Bone Tumours.

**Table 8:** Cranial and paraspinal nerve tumours

Schwannoma
Neurofibroma
Perineurioma
Hybrid nerve sheath tumour
Malignant melanotic nerve sheath tumour
Malignant peripheral nerve sheath tumour
Cauda equina neuroendocrine tumour (previously paraganglioma)

### Meningiomas

Histopathological grading is a strong prognostic factor in human meningiomas and is important for therapeutic strategies and follow-up regimes [15]. The grading system in WHO CNS5 is comparable with WHO 2016 with three malignancy grades (CNS WHO grades 1–3) based on histopathology or subtype (Table 9). Meningiomas are now regarded as a single tumour type with 15 subtypes, and the malignancy grading has been changed to a within-tumour grading regardless of subtype. Since chordoid and clear cell meningiomas have a higher risk to recur, they are assigned as grade 2. Brain-invasive meningiomas are in general associated with increased risk of recurrence and are as in WHO 2016 regarded as an atypical meningioma CNS WHO grade 2. However, assessment of brain invasion is subjective and related to sampling error, and it is also questionable whether those with benign histology and totally resected behave as grade 2 meningiomas [3]. Rhabdoid and papillary meningiomas may have a more aggressive behaviour; however, these phenotypes are now not sufficient to designate them as grade 3, and they shall now be graded as meningiomas in general [16, 27].

**Table 9:** Meningioma subtypes

Histological type	Histological malignancy grade
Meningothelial meningioma	1/2
Fibrous meningioma	1/2
Transitional meningioma	1/2
Psammomatous meningioma	1/2
Angiomatous meningioma	1/2
Microcystic meningioma	1/2
Secretory meningioma	1/2
Lymphoplasmacyte-rich meningioma	1/2
Atypical meningioma (including brain infiltrative meningiomas)	2
Chordoid meningioma	2
Clear cell meningioma	2
Anaplastic (malignant) meningioma	3

Since malignancy grading of human meningiomas is based on subjective assessment of histopathological findings, this system is suboptimal with problematic interobserver variation [37]. This is illustrated by meningiomas CNS WHO grade 1 with unexpectedly early recurrence and meningiomas CNS WHO grade 2 with long indolent clinical course without recurrence [15, 17]. Thus, WHO CNS5 endorses molecular biomarkers to refine classification and malignancy grading; however, it is not required for diagnosis if definitive histopathology of a meningioma subtype is present [16]. Advances in molecular characterisation of meningiomas have revealed several genetic aberrations and driver mutations; the most significant alterations from a clinicopathological point of view are shown in Table 10. Thus, meningiomas can be dichotomized as *NF2* (neurofibromatosis type 2) and non-*NF2*-mutated [4]. Convexity meningiomas are most often *NF2*-mutated and comprise fibroblastic and transitional phenotypes, and they are more common grade CNS WHO grade 2 and 3 [4]. Non-NF2 meningiomas are more often skull-based and comprise meningothelial and secretory phenotypes [4]. In case of aggressive atypical meningiomas and meningiomas with borderline grades 2–3 histopathology, genetic analyses have revealed that *TERTp* mutation and homozygous *CDKN2A/B* loss should be looked for and when present indicate a grade 3 tumour [16, 27]. H3K27me3 loss also indicates more aggressive behaviour [13]. DNA methylation has been shown to stratify meningiomas into methylation classes that more accurately than histopathology identify patients at high risk of recurrence [38]. Molecular classification of meningiomas based on copy number variation, point mutations, methylation, and transcriptomic and proteomic data stands out as a future diagnostic work-up of meningiomas [30].

**Table 10:** Clinicopathological relevant genetic alterations in human meningiomas

Genetic alteration	Clinicopathological significance
*NF2* mutation	Convexity meningiomas, fibrous and transitional subtypes, more often CNS WHO grade 2/3
*TRAF7* mutations	Secretory subtype
*TERT* promotor mutation	CNS WHO grade 3
*SMARCE1* mutation	Clear cell subtype
*BAP1* mutation	Rhabdoid and papillary subtypes
*CDKNA2A/B* loss	CNS WHO grade 3
H3K27me3 loss	Increased risk of recurrence
DNA methylation profiling	Methylation classes associated with increased risk of recurrence

### Mesenchymal, non-meningothelial tumours

These mesenchymal tumours are principally similar to those elsewhere in the body, and the nomenclature and histopathology of these tumours now harmonise more with the WHO classification of bone and soft tissue tumours [44]. In general, these tumours are rare in CNS, and in the revised classification, only those unique of CNS are enrolled (see Table 11). *Solitary fibrous tumour* now replaces the term “haemangiopericytoma”, a term no longer in use. They are graded on a 3-tiered scale based on a combination of mitotic counts and necroses [27].

**Table 11:** Mesenchymal, non-meningothelial tumours in CNS

**Soft tissue tumours** Fibroblastic and myofibroblastic tumours - Solitary fibrous tumour
Vascular tumours - Haemangiomas and vascular malformations - Haemangioblastoma Skeletal muscle tumours Tumours of uncertain differentiation - Intracranial mesenchymal tumour, FET::CREB fusion-positive- CIC-rearranged sarcoma - Primary intracranial sarcoma, DICER1-mutant - Ewing sarcoma
**Chondro-osseous tumours** Chondrogenic tumours - Mesenchymal chondrosarcoma - Chondrosarcoma
**Notochordal tumours** - Chordoma

### Haematolymphoid tumours

Lymphomas and histiocytic tumours are now grouped together and include those most common in CNS. Lymphomas may occur in all organs. It is therefore important to distinguish between primary and secondary manifestation of the CNS. The classification of these tumours is in line with WHO 2016. Most common primary CNS lymphoma is *diffuse large B-cell lymphoma of the CNS* (*CNS-DLBCL*), previously called “primary CNS lymphoma”.

### Germ cell tumours

Germ cell tumours of the CNS are homologue to other gonodal and extraneuraxial derivated tumours.

### Tumours of the sellar region

In WHO CNS5, *adamantinomatous* and *papillary craniopharyngiomas* are regarded as separate and distinct tumour types. *Pituitary adenomas* are diagnosed in accordance with the guidelines of WHO Blue Book of Endocrine Tumours [32].

### Metastases

Metastatic tumours to CNS are divided into those that involve brain and spinal cord parenchyma and the meninges. Regarding the latter, terms like “leptomeningeal cancer”, “neoplastic meningitis” and “(lepto)meningeal carcinomatosis” are not recommended.

### Genetic tumour syndromes of the CNS

The advent of molecular genetics has increased our knowledge of genetic tumour syndromes, so in the last WHO edition new entities are added. It is important to be aware of these syndromes, especially because of specific tumour types, clinical course and therapeutic consequences.

## Conclusions

Progress in molecular characterisation of CNS tumours provides more accurate diagnosis and prognosis, reduces the risk of sampling error and facilitates clinical decision-making. Implementation in coming and previous clinical trials may enable more tailored surgical and non-surgical treatment strategies in neuro-oncology.
